# Nox Complex signal and MAPK cascade pathway are cross-linked and essential for pathogenicity and conidiation of mycoparasite *Coniothyrium minitans*

**DOI:** 10.1038/srep24325

**Published:** 2016-04-12

**Authors:** Wei Wei, Wenjun Zhu, Jiasen Cheng, Jiatao Xie, Daohong Jiang, Guoqing Li, Weidong Chen, Yanping Fu

**Affiliations:** 1 State Key Laboratory of Agricultural Microbiology, Huazhong Agricultural University, Wuhan 430070, Hubei Province, P R China; 2 The Provincial Key Lab of Plant Pathology of Hubei Province, College of Plant Science and Technology, Huazhong Agricultural University, Wuhan, 430070, Hubei Province, P R China; 3 United States Department of Agriculture, Agricultural Research Service, Washington State University, Pullman, WA, USA

## Abstract

The NADPH oxidase complex of a sclerotial mycoparasite *Coniothyrium minitans*, an important biocontrol agent against crop diseases caused by *Sclerotinia sclerotiorum*, was identified and its functions involved in conidiation and mycoparasitism were studied. Gene knock-out and complementary experiments indicated that *CmNox1*, but not *CmNox2*, is necessary for conidiation and parasitism, and its expression could be significantly induced by its host fungus. CmNox1 is regulated by CmRac1-CmNoxR and interacts with CmSlt2, a homolog of *Saccharomyces cerevisiae* Slt2 encoding cell wall integrity-related MAP kinase. In Δ*CmNox1*, CmSlt2-GFP fusion protein lost the ability to localize to the cell nucleus accurately. The defect of conidiation in Δ*CmRac1* could be partially restored by over-expressing *CmSlt2*, indicating that CmSlt2 was a downstream regulatory factor of CmNox1 and was involved in conidiation and parasitism. The expressions of mycoparasitism-related genes *CmPks1*, *Cmg1* and *CH1* were suppressed in the knock-out mutants of the genes in CmNox1-CmSlt2 signal pathway when cultivated either on PDA. Therefore, our study infers that CmRac1-CmNoxR regulates CmNox1-CmSlt2 pathway in regulating conidiation and pathogenicity of *C. minitans.*

*Sclerotinia sclerotiorum* (Lib.) de Bary is a significant necrotrophic pathogen that could infect more than 400 plant species worldwide and cause huge economic losses every year^1^,^2^. *Coniothyrium minitans* is an important mycoparasite of *Sclerotinia* spp.; it could parasitize and destroy both sclerotium and hypha of its hosts. It is an effective agent for controlling crop diseases caused by *S. sclerotiorum*^3^,^4^,^5^. Like its hosts, *C. minitans* also occurs widely^6^. It shares the same growing season as its hosts, and its growth and proliferation are tightly related to the hosts. Without host fungi, *C. minitans* stays dormant for seasons in soil^7^. *C. minitans* is one of the commercialized fungal agents for biological control of crop diseases^8^.

Understanding the parasitism and conidiation of *C. minitans* at molecular level could help us to utilize this biological control agent more efficiently. Studies indicated that the primary cell wall degrading enzymes secreted by *C. minitans* are β-1,3 glucanase (encoded by *Cmg1*) and chitinase (encoded by *CH1*). The expression level of *Cmg1* was enhanced when *C. minitans* parasitized *S. sclerotiorum,* indicating that β-1,3 glucanase played important roles in the parasitical process^9^. *C. minitans* also could secrete antifungal substances and degrade oxalic acid, an essential pathogenicity factor of *S. sclerotiorum*^10^,^11^. The parasitism of *C. minitans* is likely very complicated^12^, and shares some signal transduction pathways with conidiation. Fungal cell wall integrity-associated MAP kinase cascade, fatty acid beta-oxidation, reactive oxygen and nitrogen species, and possibly other unknown pathways in peroxisomes are required for both conidiation and mycoparasitism of *C. minitans*^13^,^14^. Recent research revealed that *C. minitans* can regulate ambient pH by degrading oxalic acid to facilitate mycoparasitism of *S. sclerotiorum*^15^,^16^. *C. minitans* requires a large amount of L-arginine during conidiation and L-arginine-derived nitric oxide was likely to be involved in conidiation with cyclic GMP functions as a second messenger^17^,^18^. Qin *et al.* found that phosphoribosylamidotransferase is essential for conidiation of *C. minitans* via adenosine related molecules, and *C. minitans* is able to obtain adenosine or related components from its host during parasitization^19^.

The NADPH oxidase complexes are conserved and play important roles in the life cycle of filamentous fungi^20^. In rice blast pathogen *Magnaporthe oryzae*, NADPH oxidase-derived reactive oxygen species (ROS) is essential for pathogenicity^21^, and further study found that NADPH oxidases are necessary for septin-mediated reorientation of the F-actin cytoskeleton to facilitate cuticle rupture and plant cell invasion^22^. NADPH oxidases are involved in sclerotial formation and pathogenicity of necrotrophic fungal pathogen *Botrytis cinerea* and *S. sclerotiorum*^22^,^23^,^24^,^25^, and fusion of conidial anastomosis tubes of *B. cinerea*^26^. ROS is critical in maintaining a mutualistic interaction between *Epichloë festucae* and perennial ryegrass^27^,^28^,^29^,^30^,^31^. In saprophytic fungi, such as *Aspergillus* spp., *Neurospora crassa* and *Podospora anserina*, NADPH oxidases are required for growth, cell differentiation, conidiation, and sexual reproduction^32^,^33^,^34^,^35^,^36^. *Trichoderma harzianum* NADPH oxidases are involved in the antagonism against *Pythium ultimum*^37^.

Besides NADPH oxidases mitogen-activated protein kinases (MAPKs) also play critical roles in pathogenicity and in fungal development^38^,^39^,^40^,^41^,^42^,^43^,^44^,^45^,^46^,^47^. In endophytic fungi, a stress-activated mitogen-activated protein kinase (sakA) of *E. festucae* is essential to maintain mutualistic symbiosis with perennial ryegrass. Deletion of sakA converted this endophytic fungus to a pathogen of its host^48^. MAP kinase cascade also is involved in the mycoparasitism and development of hyperparasitic fungi, such as *C. minitans*, *Stachybotrys elegans* and *T. artoviride*^13^,^49^,^50^. The phenotype of Δ*sakA* mutants are very similar to that of mutants whose genes in the Nox complex were disrupted, suggesting that there is a possible link between ROS signal and MAPK signal pathway on the maintenance of mutualistic symbiosis^29^. Similar phenomena were observed in tangerine pathogen *Alternaria alternate*^51^; Medina-Castellanos *et al.* found that extracellular ATP could promote the Nox1-derived ROS and activate a MAPK pathway in *T. artoviride*^52^. Recently, Jaimes-Arroyo *et al.* found that SrkA kinase could regulate stress responses and development in *A. nidulans,* and H_2_O_2_ could induce mitochondrial fragmentation and relocalize SrkA at the presence of SakA^53^.

Previously, we investigated a fungal cell wall integrity-associated MAP kinase cascade in *C. minitans* and found that this cascade was required for conidiation and mycoparasitism^13^. In this study, we analyzed the function of NADPH oxidases (Nox1/Nox2) complex of *C. minitans*, and found that *CmNox1* played critical roles in conidiation and mycoparasitism, but not *CmNox2*. In *C. minitans*, CmNoxR interacts with CmRac1 to activate CmNox1, CmNox1 could also interact with CmSlt2 and thus adjust its location to the cell nucleus, and deliver the signal of conidiation and mycoparasitism.

## Results

### NADPH oxidases (Nox1/Nox2) in *C. minitans*

Two NADPH oxidase genes, *CmNox1* and *CmNox2*, were isolated from *C. minitans*. The deduced amino acid sequence of *CmNox1* (GenBank Accession No: KJ596434) shows high similarity to Nox1 homologs from other filamentous fungi, including *Alternaria alternate* (AaNox1, BAK52527.1, 89% identity), *Curvularia lunata* (ClNOX1, AHC53982.1, 89% identity), and *Pyrenophora tritici-repentis* (PrNOX1, XP_001935118.1, 88% identity). *CmNox2* (GenBank Accession No: KJ596435) is highly similar to Nox2 homologs from other filamentous fungi. The multiple alignment analysis showed that both CmNox1 and CmNox2 contained NOX family signature regions. Phylogenetic analysis of NADPH oxidases in several fungi placed CmNox1 and CmNox2 homologs into two different clades based on the amino acid sequences (Supplementary Figure S1).

### *CmNox1* is essential for ROS production and conidiation

To study the function of *CmNox1* in *C. minitans*, a replacement vector p3300neoCmNox1 (see Supplementary Figure S2a) was constructed and transformed into strain ZS-1 to disrupt *CmNox1*. Twenty transformants were obtained, and three of which, Δ*CmNox1-1*, Δ*CmNox1-6*, and Δ*CmNox1-107* were selected randomly as candidates for further analyses. Furthermore, a complement vector pNox1 was transformed into mutant Δ*CmNox1-6*. The deletion and complementary events were confirmed by RT-PCR (see Supplementary Figure S2b) and Southern blot analysis (see Supplementary Figure S2c). Using the same methods, a replacement vector p3300neoCmNox2 was constructed (see Supplementary Figure S2a) and transformed into strain ZS-1 to disrupt *CmNox2*. Three deletion mutants Δ*CmNox2-20,* Δ*CmNox2-323* and Δ*CmNox2-347* were also confirmed by Southern blot analysis.

**Figure 1:**
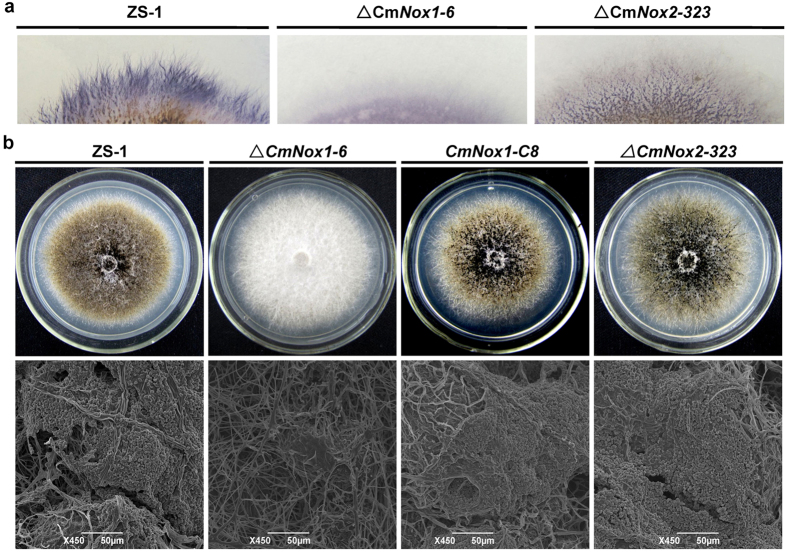
Production of superoxide and conidia of *Coniothyrium minitans CmNox1* and *CmNox2* mutants. (**a**) Detection of superoxide using colony staining with NBT solution (0.05% w/v, 30 min) of cultures of *C. minitans* wild-type strain ZS-1, *CmNox1* deletion mutant Δ*CmNox1-6*, *CmNox2* deletion mutant Δ*CmNox2-323* on PDA. (**b**) Pycnidial and conidial production of *C. minitans* wild-type strain ZS-1, *CmNox1* deletion mutant Δ*CmNox1-6*, complementary transformant *CmNox1-C8*, and *CmNox2* deletion mutant Δ*CmNox2-323* 12 days after incubation on PDA. Compared to ZS-1, *CmNox1-C8* and Δ*CmNox2-323*, Δ*CmNox1-6* lost the ability to produce pycnidia.

Colony staining with NBT solution showed that superoxide production was decreased significantly in Δ*CmNox1-6*, compared to Δ*CmNox2-323* and the wild-type strain ZS-1 (Fig. 1a). The conidiation of the wild-type strain ZS-1, *CmNox1* deletion mutants, *CmNox1* complemented mutants, and *CmNox2* deletion mutants were determined after incubating for 15 days on PDA (Fig. 1b). *CmNox1* deletion mutants completely lost the ability to produce conidia (Table 1). In contrast, the wild-type strain ZS-1, *CmNox1* complemented mutants and *CmNox2* deletion mutants were normal in conidiation under the same condition (Table 1). Unlike strain ZS-1 and Δ*CmNox2-323*, which could form matured pycnidia and conidia, Δ*CmNox1-6* could only form a few pycnidial primordia that could not further develop to mature pycnidia, and no conidium was produced (Fig. 1b). These data indicated that *CmNox1*, but not *CmNox2*, played significant roles in conidiation and production of superoxide. The experiments also suggested *CmNox1* and *CmNox2* are not essential for hyphal growth of *C. minitans* on PDA.

### *CmNox1* is essential to parasitize sclerotia of *S. sclerotiorum*

**Figure 2:**
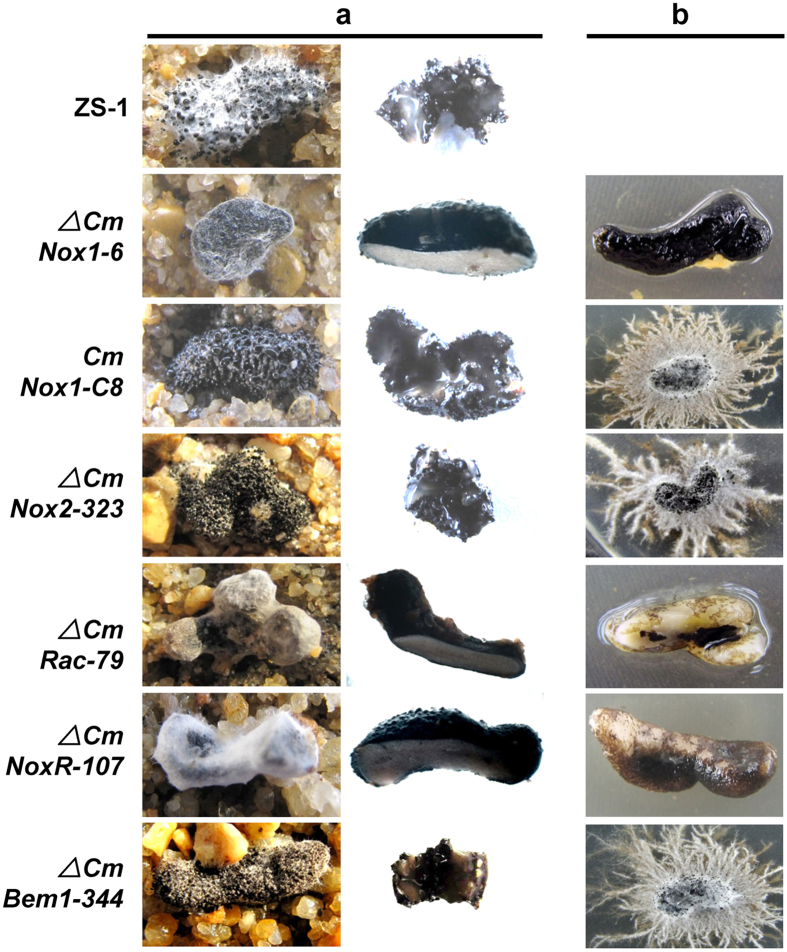
Parasitism of *C. minitans* mutants to sclerotia of *S. sclerotiorum*. (**a**) Microscopic observation of the surface and interior of sclerotia infected by *C. minitans*. (**b**) Colony formed by all inoculated sclerotia. All inoculated sclerotia were surface sterilized and then placed on hygromycin (50 μg/ml) amended PDA for 7 days.

To determine whether *CmNox1* is related to sclerotial parasitizing, the parasitic ability of *CmNox1* mutants and the other strains to sclerotia of *S. sclerotiorum* were examined. The hyphae of Δ*CmNox1-6* was inoculated on to sclerotia and incubated for 30 days at 20 °C, and no pycnidia and conidia were observed on either the surface or interior of the sclerotia (Fig. 2a, Table 1). The inoculated sclerotia were surface sterilized and then incubated on PDA containing 50 μg/ml hygromycin, and no *C. minitans* colony emerged from the sclerotia, suggesting that Δ*CmNox1-6* did not invade the inner of sclerotia (Fig. 2b, Table 1). Meanwhile, *CmNox1-C8,* Δ*CmNox2-323* and the wild-type strain ZS-1 could degrade sclerotia and produce mature pycnidia there. The results demonstrated *CmNox1* played a significant role in sclerotial mycoparsitism.

### Expression of *CmNox1* is highly induced by *S. sclerotiorum*

**Figure 3:**
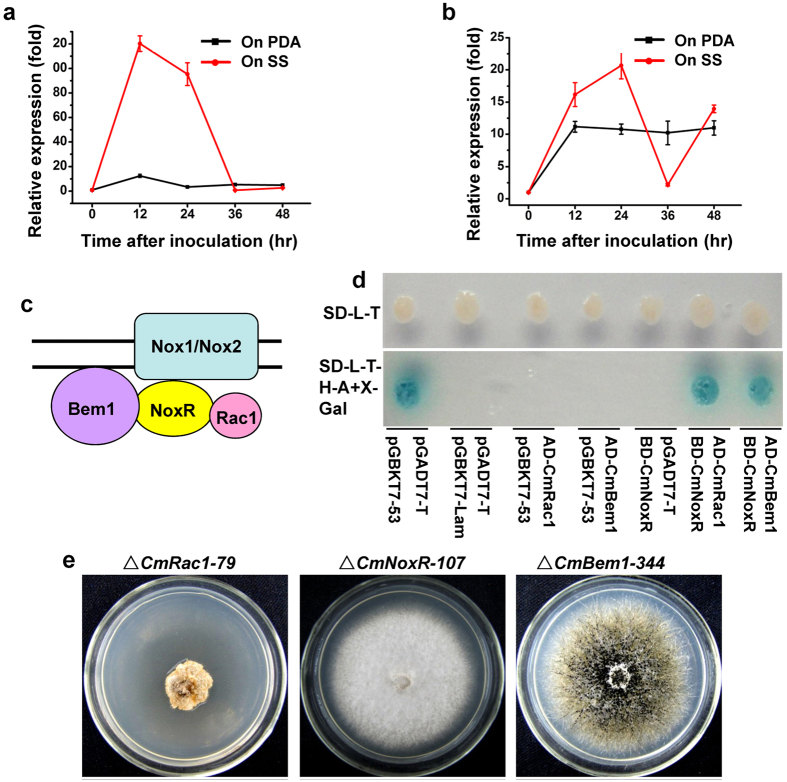
NADPH oxidase complexes in *C. minitans.* Expression analysis of *CmNox1* (**a**) and *CmNox2* (**b**). Total RNA was isolated from mycelia of ZS-1 cultured alone or dual-incubated with *S. sclerotiorum* on PDA, and used for cDNA synthesis. The transcript level of *CmActin* was used to normalize different samples. Bars represent means and standard deviations (three replications). (**c**) Schematic drawing showing the NADPH oxidase complexes in *C. minitans.* (**d**) Yeast two-hybrid assay of interaction between CmNoxR, CmBem1, and CmRac1. (**e**) Colony morphology of *CmRac1* deletion mutant Δ*CmRac1-79*, *CmNoxR* deletion mutant Δ*CmNoxR-107* and *CmBem1* deletion mutant Δ*CmBem1-344* grown on PDA at 20 °C for 12 days.

When dual cultured with *S. sclerotiorum*, the expression of *CmNox1* was highly induced. Compared to growing on PDA, the expression of *CmNox1* peaked at 12 hpi, and the high expression was maintained till 24 hpi, and then sharply declined to undetectable level at 36 hpi (Fig. 3a). This phenomenon suggested that *CmNox1* played an important role in parasitism. Expression of *CmNox2* also was up-regulated slightly by *S. sclerotiorum* at 12 hpi and 24 hpi, but compared to culture on PDA, the induction was much lower (Fig. 3b). The results showed that *S. sclerotiorum* could induce the expression of *CmNox1* and *CmNox2*, with stronger induction to *CmNox1*.

### CmRac1 interacts with CmNoxR together to regulate CmNox1 and to control conidiation in *C. minitans*

It is reported that Bem1 and Cdc24 are components of the NADPH oxidase complex in filamentous fungi, and NoxR interacting with RacA *in vitro* together regulates ROS production and control hyphal branching and patterning in *E. festucae*^27^,^29^. Since *CmNox1* is involved in the production ROS in *C. minitans*, we identified *C. minitans* homologs of NoxR, GTPase Rac1 and Bem1, named *CmNoxR* (GenBank Accession No: KJ596436), *CmRac1* (GenBank Accession No: KJ596436) and *CmBem1*, respectively. The interaction between CmRac1 and CmNoxR, CmBem1 and CmNoxR were confirmed by Yeast two-hybrid (Fig. 3c,d).

To investigate the role of *CmNoxR, CmBem1* and *CmRac1* in *C. minitans*, replacement vector for *CmNoxR* (p3300neoCmNoxR), *CmRac1* (p3300neoCmRac1), and *CmBem1* (p3300neoCmBem1) were constructed and transformed into ZS-1. The *CmNoxR* deletion mutants (Δ*CmNoxR-5* and Δ*CmNoxR-107*), *CmBem1* deletion mutant (Δ*CmBem1-344*) and *CmRac1* deletion mutant (Δ*CmRac1-79*) were obtained and confirmed by RT-PCR. In contrast to the wild-type strain ZS-1, the *CmNoxR* deletion mutants and the *CmRac1* deletion mutant lost the ability to produce conidia and to parasitize *S. sclerotiorum*. In addition to the conidiation and parasitizing phenotype, growth rate also was reduced significantly in the *CmRac1* deletion mutant. The growth rate of Δ*CmRac1-79* was less than 0.6 mm/day on average on PDA, while it was about 3mm/day for strain ZS-1 under the same conditions. However, there was no significant difference in the growth rate between *CmNoxR* deletion mutants and ZS-1, and the deletion mutant displayed a phenotype similar to *CmNox1* deletion mutant (Fig. 3e, Table 1). Δ*CmBem1-344* shared the same phenotype as strain ZS-1. It suggests that CmRac1and CmNoxR are required to activate CmNox1 to adjust conidiation and parasitism, while CmBem1 is not involved.

### CmSlt2 is CmNox1 effector required for conidiation and parasitism

**Figure 4:**
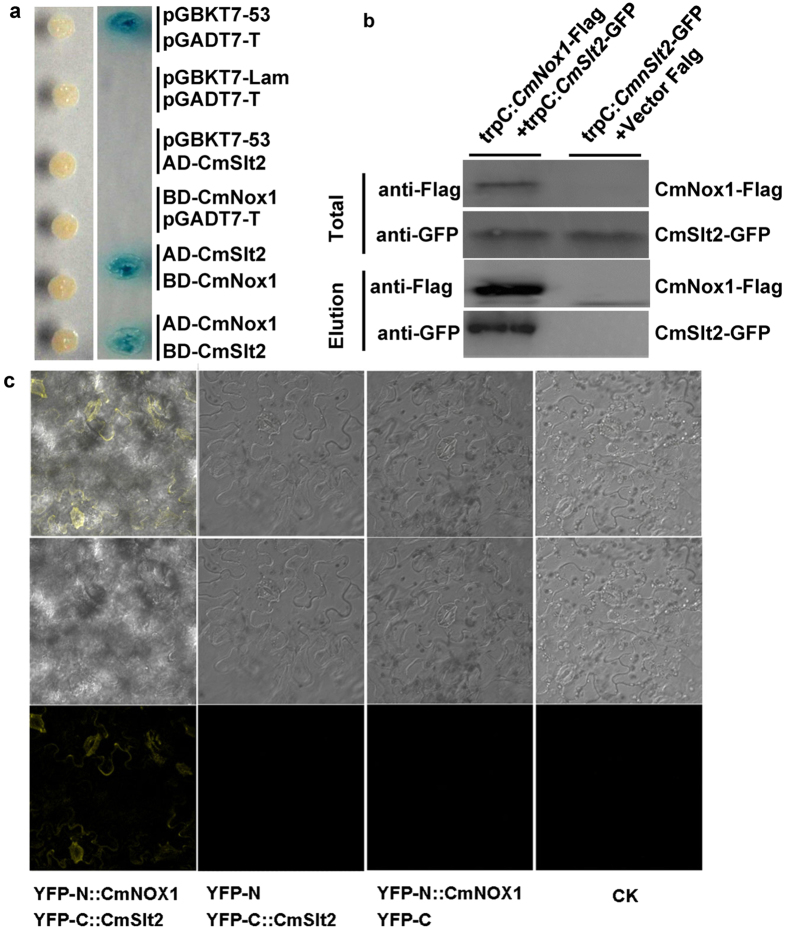
Interaction of CmNox1 with CmSlt2. (**a**) Yeast two-hybrid assay of interaction between CmNox1 and CmSlt2. (**b**) Co-IP assays. Western blots of total proteins and proteins eluted from anti-Flag agarose from transformant (*CmSlt2*-GFP and vector Flag) and transformant (*CmSlt2*-GFP and *CmNox1*-Flag) were detected with anti-Flag or anti-GFP antibodies. (**c**) Bimolecular fluorescence complementation (BiFC) analysis of the interaction between CmNox1 and CmSlt2 tagged with YFP in *Nicotiana benthamiana*.

To understand the mechanism of CmNox1-mediated conidiation and parasitism in *C. minitans,* we further investigated the functional relationship between CmNox1 and CmSlt2. We reported previously that *CmSlt2* (cell wall integrity-related MAP kinase) is essential for conidiation of *C. minitans*. Δ*CmSlt2* lost the ability to produce pycnidia and conidia, and had dramatically reduced sclerotial mycoparasitism^13^. The phenotype of Δ*CmSlt2* was similar to that of Δ*CmNox1*, thus CmSlt2 and CmNox1 might have somewhat interaction directly or indirectly. To identify whether CmSlt2 interact with CmNox1, full length gene of *CmSlt2* and *CmNox1* were cloned into yeast two-hybrid vector pGADT7 and pGBDT7 to test the possible interaction, and the results showed that CmSlt2 could interact with CmNox1 (Fig. 4a). Furthermore, we generated fusions of CmSlt2 and CmNox1 with N- and C-terminal domains of YFP and assessed their interaction in tobacco (*Nicotiana benthamiana*) using bimolecular fluorescence complementation (BiFC) (Fig. 4c). To confirm this interaction in *C. minitans,* a transformant with TrpC-*CmNox1*-Flag and TrpC-*CmSlt2*-GFP and a transformant with TrpC-*CmSlt2*-GFP and Vector-Flag in ZS-1 were constructed. In Western blot analysis with total protein, the anti-Flag and anti-GFP antibodies detected a 65-kDa and an 80-kDa band, respectively. In proteins eluted from anti-Flag agarose, the 80-kDa CmSlt2-GFP band was detected with an anti-GFP antibody in transformant with *CmSlt2* and *CmNox1*, but not in transformant with *CmSlt2* only (Fig. 4b). The results showed that CmSlt2 and CmNox1 interacted directly in *C. minitans*.

**Figure 5:**
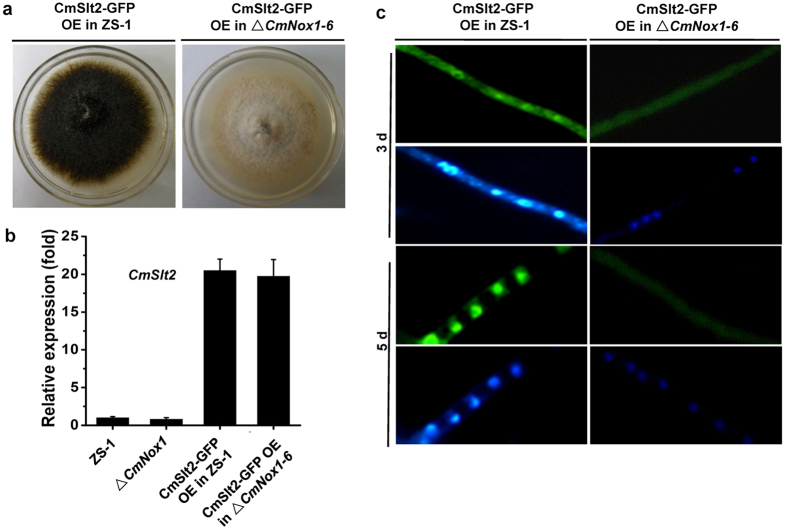
CmNox1 controls the nucleus location of CmSlt2. (**a**) Colony morphology of *CmSlt2-*GFP-overexpressed ZS-1 and Δ*CmNox1-6.* (**b**) Relative expression of *CmSlt2* in *CmSlt2-*GFP-overexpressed ZS-1 and Δ*CmNox-1-6.* The expression of ZS-1 was set as level one. (**c**) CmSlt2-GFP nuclear localization in *CmSlt2-*GFP-overexpressed ZS-1 and Δ*CmNox1-6.*

We expressed the *CmSlt2*-GFP fusion in the wild-type strain ZS-1 and Δ*CmNox1*, and found that over-expressing *CmSlt2*-GFP could not rescue the deficiency of conidiation and parasitism in Δ*CmNox1* (Fig. 5a,b). In the wild-type strain ZS-1, CmSlt2 was located in the cytoplasm during the hyphal growth period (3-day-old hyphae), and was located in the nucleus in 40% of the hyphae at the later stage of conidiation (5-day-old hyphae) observed under a fluorescence microscope; While in Δ*CmNox1*, no fluorescence signal of *CmSlt2*- GFP was observed in the nucleus at any stages (Fig. 5c). The results suggested that CmSlt2 was a downstream regulatory factor of CmNox1 and was involved in conidiation and parasitism.

### Over expressing *CmSlt2* in Δ*CmRac1* could partially restore conidiation and parasitism

**Figure 6:**
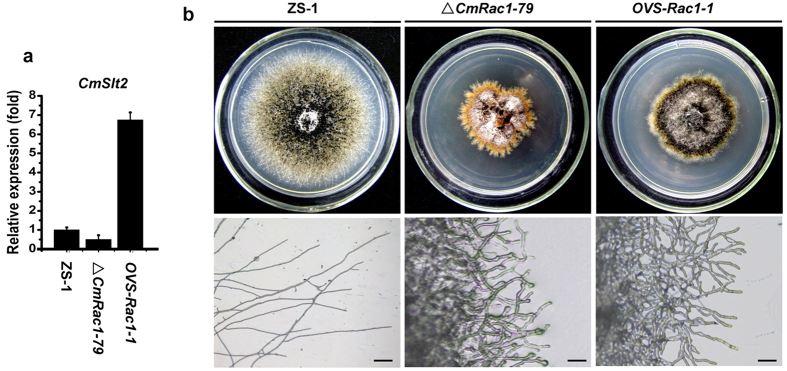
Over-expression of the MAP kinase gene *CmSlt2* partially restored the conidiation of Δ*CmRac1-79*. (**a**) The relative expression of *CmSlt2* in the wild type strain ZS-1, Δ*CmRac1-79* and *OVS-Rac1-1* (*CmSlt2* over-expressing in *ΔCmRac1-79*). (**b**) Comparison of colony morphology and tip hyphae branching pattern among the wild-type strain ZS-1, Δ*CmRac1-79*, and *OVS-Rac1-1* grown on PDA medium at 20 °C for 20 days.

Since CmRac1 interacts with CmNoxR together in regulating CmNox1, and CmNox1 mediates the localization of CmSlt2 in nucleus, we suspected that CmRac1 is likely to regulate the expression of *CmSlt2*. qRT-PCR analysis showed that the expression of *CmSlt2* was obviously decreased in Δ*CmRac1* by 0.5 fold, compared to the wild-type strain (Fig. 6a). We fused *CmSlt2* with a trpC promoter from *Aspergillus* and transformed into Δ*CmRac1*, and found that over-expressing of *CmSlt2* could partially restore conidiation and parasitic ability of Δ*CmRac1* (Fig. 6b, and Table 1), in spite of no significant improvement of the hyphal growth.

### The expression of *CmPks1* was suppressed in CmNox1 signal pathway mutants

**Figure 7:**
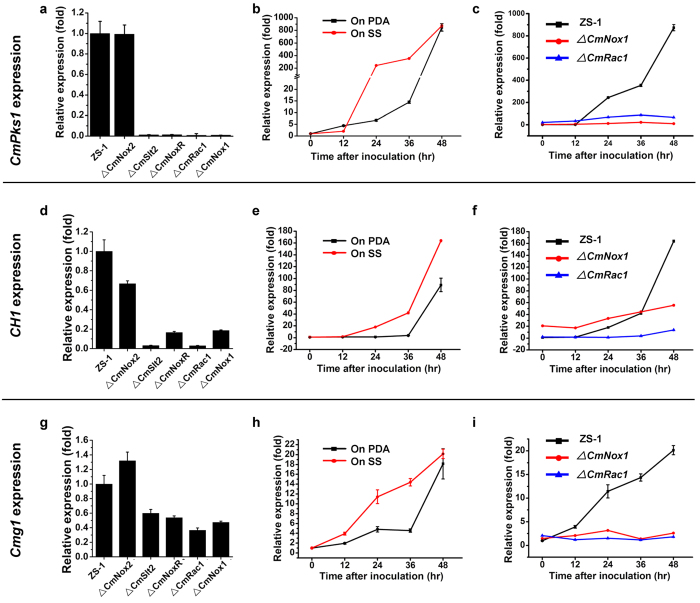
Gene expression analysis in the wild-type strain ZS-1 and deletion mutants of *C. minitans*. Relative transcript accumulations of *CmPks1* (**a**), *CH1* (**d**) and *Cmg1* (**g**) detected with qRT-PCR amplification in ZS-1, Δ*CmNox2*, Δ*CmSlt2*, Δ*CmNoxR*, Δ*CmRac1* and Δ*CmNox1* after growing on PDA for 96 hr. The expression of ZS-1 was set as level one. Relative transcript patterns of *CmPks1* (**b**), *CH1* (**e**) and *Cmg1* (**h**) in ZS-1 after contacting with *S. sclerotiorum* (red) or growing on PDA (black) for 0–48 hr. The gene expression of ZS-1 inoculated in plate at 0 hr was set as level one. Relative transcript patterns of *CmPks1* (**c**), *CH1* (**f**) and *Cmg1* (**i**) genes in ZS-1 (black), Δ*CmNox1* (red) and Δ*CmRac1* (blue) after growing on PDA for 0–48 hr. The gene expression of ZS-1 at 0 hr was set as level one. The relative level of transcript was calculated by the comparative Ct method. The level of *CmActin* transcript was used to normalize different samples. Bars represent means and standard deviations (three replications).

*C. minitans* produces dark pigment and black pycnidia during the late stage of growth. However, all Δ*CmRac1*, Δ*CmNoxR*, Δ*CmNox1*, and Δ*CmSlt2* produce little dark pigment, and colonies were whitish. It is likely that the expression of *CmPks1*, a melanin biosynthesis associated polyketide synthase-encoding gene, was suppressed in those mutants. The transcript profile of *CmPks1* was monitored by qRT-PCR, and the result showed that the expression of *CmPks1* peaked at 96 hpi in the wild type ZS-1 and was significantly suppressed in Δ*CmNoxR*, Δ*CmRac1*, Δ*CmNox1* and Δ*CmSlt2*, whereas there was no obvious difference in the transcript level between Δ*CmNox2* and ZS-1 (Fig. 7a). Further experiments proved that the expression of *CmPks1* in ZS-1 was enhanced by 300–400 fold when dual cultured with *S. sclerotiorum* than cultured on PDA alone at 24 hpi and 36 hpi (Fig. 7b), while it could not be induced in Δ*CmRac1* and Δ*CmNox1* (Fig. 7c). These results indicated that the expression of *CmPks1* could be induced at the early stages of interaction with *S. sclerotiorum*, while could not in all of the CmNox1 signal pathway mutants either cultured on PDA or interacted with *S. sclerotiorum*. It suggested that the *CmNox1* signal pathway is involved in regulating the expression of *CmPks1*.

### The CmNox1 signal pathway is involved in regulating cell wall degrading enzyme genes

The parasitic ability of all mutants in CmNox1 signal pathways decreased obviously and it is also reported that the cell wall degrading enzyme *Cmg1* (β-1,3 glucanase-encoding gene) and *CH1* (chitinase gene) play important roles in the process of parasitizing sclerotia of *S. sclerotiorum*^9^. Our results of qRT-PCR demonstrated that the transcript levels of both *Cmg1* and *CH1* in ZS-1 were enhanced significantly at 24 hpi and 36 hpi when dual cultured with *S. sclerotiorum* (Fig. 7e,h), while were suppressed in Δ*CmNoxR*, Δ*CmRac1*, Δ*CmNox1* or Δ*CmSlt2* when incubated on PDA compared to that in ZS-1 (Fig. 7d,f,g,i). The results indicated that *Cmg1* and *CH1* were involved in parasitism and could be regulated by the CmNox1 signal pathway.

## Discussion

The mycoparasitism system of *C. minitans*/*S. sclerotiorum* is unique and important to probe fungi and fungi interaction. In this study, the role of NADPH oxidases complex of *C. minitans* on growth, conidiation and mycoparasitism were studied. We found that *CmNox1*, but not *CmNox2*, played essential roles on conidiation and mycoparasitism. Furthermore, we found that CmRac1 could interact with CmNoxR, and CmNox1 could interact with CmSlt2. Expression of *CmSlt2* in Δ*CmRac1* could partially restore the conidiation and parasitism. These findings broadened and strengthened our knowledge of mycoparasitism, and supplied a possible link between Nox complex signal and MAPK cascade signal in fungi.

Fungal Nox isoforms have different roles in fungi lifecycle. In *M. oryzae*, both Nox1 and Nox2 are essential for pathogenicity^21^. Nox1 is required for penetration, hyphal elongation, and Nox2 is required for assembly of a toroidal F-actin network during the penetration peg formation^22^; In *S. sclerotiorum*, Nox1 is required for virulence, oxalic acid production and sclerotial development, while, Nox2 might be involved in sclerotial development^24^. In *P. anserine*, *PaNox1* mutants are impaired in the differentiation of fruiting bodies from their progenitor cells, and deletion of the *PaNox2* specifically blocked ascospore germination^33^,^54^. New Nox proteins were also identified from *P. anserine*, a Nox isoform Nox3 was found to play a minor role^36^. In *N. crassa*, Nox1 is required for female sterility, and involved in asexual development and hyphal growth, while Nox2 might be involved in ascospore germination^34^. In *E. festucae*, both Δ*NoxA* and Δ*NoxB* mutants could produce conidia, while NoxA, but not NoxB, is essential for hyphal polarized growth and hyphal funsion, NoxB did not affect conidiation^29^. In *Sordaria macrospora*, Nox1 is required for fruiting body formation, normal hyphal growth, and hyphal fusion, while Nox2 is involved in strict melanin-dependent ascospore germination^55^. In *C. purpurea*, CpNox1 is essential for infection, but CpNox2 is not essential for infection. Interestingly, Δ*cpnox2* mutants converted endophytic lifestyle to pathogenic lifestyle, and CpNox2 functioned in the infection process and moderates damage to the host^56^. In this study, the Nox1 of *C. minitans* is essential for mycoparasitism, conidiation and pigmentation, while deletion of Nox2 did not affect these bioprocesses. However, how Nox1 regulates these bioprocesses is necessary to be unraveled.

Rac1, a member of the Rho-family GTPases, widely exists in eukaryotes and in fungi as well. Sequence alignment of RAC1 homologs from fungi revealed significant conservation in amino acid composition and RAC1 from *M. grisea* could rescue the conidiation, parasitization and growth in Δ*Rac1* mutantof *C. minitans* (data not shown). Fungal Rac1 is crucial for the growth, virulence and development in many fungi, such as *M. grisae*, *U. maydis*, *A. fumigatus*, *Claviceps purpurea* and *F. graminearum*^57^,^58^,^59^,^60^,^61^. In *M. grisea,* MgRac1 is essential for conidiogenesis, and contributes to the formation of appressorium and pathogenicity through activating its downstream factors: the PAK kinase Chm1 and NADPH oxidases^58^. In *Candida albicans,* Rac1 is an upstream regulatory factor of the MAP kinase Cek1 and Mkc1, but the control mechanism is still unclear^62^. In this study, the qRT-PCR assay demonstrated that the expression level of *CmSlt2* was obviously decreased in Δ*CmRac1*. When *CmSlt2* was over expressed in Δ*CmRac1*, the conidiation could be partially restored, further confirming that Rac1was an upstream regulatory factor of MAPK cascade signal pathway.

The fact that the Nox complex mutants and the MAPK cascade pathway mutants of fungi sharing highly similar phenotypes suggests that these two signal pathways are cross-linked. This study provides further evidence supporting this cross-link since CmNox1 could interact with CmSlt2, and CmRac1 could interact with CmNoxR. Lalucque *et al.* reported that PaNox1 acted on the PaASK1/PaMKK1/PaMpk1 MAPK module by promoting nuclear translocation of the PaMpk1 MAP kinase in *P. anserina*^63^. More recently, H_2_O_2_ was found to induce the relocalization of a putative MAPK-activated protein kinase SrkA to nuclei and mitochondria under the presence of SakA in *A. nidulans*^53^. We further found that CmNox1 could regulate the nucleus location of a cell wall integrity-associated MAP kinase (CmSlt2). In ∆*CmNox1*, CmSlt2 could not move into fungal nuclei, suggesting that Nox complex-derived ROS may function on the localization of CmSlt2.

The Nox complex signal and MAPK cascade signal affect fungal gene expression globally to regulate complicated processes in physiology, pathogenicity and development. In *S. macrospora*, Nox1 affects the expression of genes involved in cytoskeleton remodeling, hyphal fusion, metabolism, and mitochondrial respiration^55^. In *P. anserine*, the expressions of 15% genes were modified in Δ*PaMpk1*, Δ*PaMpk2* and Δ*PaNox1*, and about 1000 genes were regulated similarly in these three mutants^64^. We attempted to explain how the Nox complex signal and MAPK cascade signal regulate mycoparasitism by monitoring the expression levels of *CmPks1*, *Cmg1* and *CH1* in *C. minitans*. We found that expression of these genes was obviously enhanced when interacted with its host fungus *S. sclerotiorum*., while could not be induced in Δ*CmNox1*, Δ*CmRac1* and Δ*CmSlt2*, indicating that these three genes were regulated by the Nox complex signal and MAPK cascade signal pathways, thus to affect parasitism. Mycoparasitism of *C. minitans* is a very complicated process. There are many other genes that are also involved in the mycoparasitism and remain to be investigated.

Briefly, we analyzed the function of Nox complex on mycoparasitism and conidiation of *C. minitans*, and we found that it is *CmNox1*, but not *CmNox2*, that is required for mycoparasitism and conidiation. We further found that CmNox1 interacts with MAP kinase CmSlt2 and effects the location of CmSlt2. Our finding suggests that Nox complex signal pathway and MAPK cascade signal pathway are cross-linked via CmNox1.

## Methods

### Strains and cultural conditions

The *C. minitans* wild-types train ZS-1 (CCAM 041057) was isolated from garden soil at Zhushan County, Hubei Province, P R China^67^. Strain Ep-1PNA367 was a virulent and virus-free strain of *S. sclerotiorum*, derived from a single ascospore of the hypovirulent strain Ep-1PN^67^. The strains used in this research were maintained and cultured on PDA at 20–22 °C and stored in PDA slants at 4 °C^17^,^67^. Cultures for genomic DNA and RNA isolation were conducted on PDA at 20–22 °C for 4 d. Conidia were prepared from 15-day-old cultures grown on PDA. The selective PDA was supplemented with 50 μg/ml of hygromycin B (Sigma) or 80 μg/ml of G418 (Sigma), depending on the selection marker in the plasmid vector.

### Analysis of colony morphology, growth rate, conidiation and parasitic ability

Colony morphology was observed on PDA after incubating at 20 °C for 12 days. Observation of the pycnidial formation was performed by following the method described^14^. To characterize the biological properties of the mutants, growth rate, conidial production and parasitic ability were examined as described^13^,^14^. For all the transformants obtained, three individuals were examined (Table1).

### ROS detection assay

Production of superoxide was evaluated with NBT using the method modified from Chen *et al.*^58^. The wild-type ZS-1, mutants Δ*CmNox1* and Δ*CmNox2* were grown on PDA for 7 days. Mycelia were incubated in 0.05 M sodium phosphate buffer, pH 7.5, containing 0.05% (w/v) NBT (Sigma-Aldrich). After 1 h of incubation, the culture was fixed in ethanol to stop the reaction. The stained sample was examined with a compound microscope at ×400 magnification.

### Isolation of CmNox1/CmNox2

A PCR primer pair 1F1/1R1 (Table S1) was designed based on the *C. minitans* genome database. *CmNox1* was amplified from the ZS-1 genomic DNA by a 32-cycle PCR reaction (94 °C, 1 min; 58 °C, 1 min; 72 °C, 2 min), followed by a 10 min extension at 72 °C. The PCR product was cloned into the pMD18-T vector (TaKaRa) and confirmed by DNA sequencing. The cDNA of *CmNox1* was isolated by RT-PCR from total RNA of *C. minitans* with the primer pair 1F1 and 1R1, followed by cloning into the pMD18-T vector and direct DNA sequencing. The same method was used to clone *CmNox2* in *C. minitans* with the primer pair 2F1/2R1 (Table S1).

### Vector construction and Agrobacterium-mediated transformation

The *CmNoxR*, *CmRac1*, *CmNox1*, *CmNox2* and *CmBem1* replacement constructs were generated according to Qin *et al.*^19^. The *CmNox1* (primer pairs 9F1/9R1 and 10F1/10R1), *CmNox2* (primer pairs 11F1/11R1 and 12F1/12R1), *CmNoxR* (primer pairs 13F1/13R1 and 14F1/14R1), *CmRac1* (primer pairs 15F1/15R1 and 16F1/16R1), and *CmBem1* (primer pairs 17F1/17R1 and 18F1/18R1) deletion constructs were made by PCR amplification of the 5′- and 3′- flanks of the respective ORFs (0.8 –1.2 kb) with genomic DNA of the wild-type strain ZS-1as template. The fragments were cloned upstream and downstream of *hph* cassette in pMD18 respectively, and then the structures were cloned into the corresponding sites of vector pneo-P3300III, which carries a neomycin resistance gene cassette (neo) as the second selection marker.

In order to construct the complementary vector of *CmNox1*, a 2.7 kb fragment containing the native promoter and ORF of *CmNox1* was amplified by PCR with primer pair 19F1/19R1 (Table S1) and cloned into vector pneoP3300. The complementary mutant CmNox1-C was generated by introducing the vector into Δ*CmNox1-6*, followed by screening for neomycin resistance and RT-PCR confirmation.

cDNA of *CmSlt2* was amplified by RT-PCR with primer pair 20F/20R (Table S1) and cloned into the *Hin*dIII/*Sma*I sites of pCIT vector, which contains the trpC promoter and terminator, and then the gene cassette was cloned into vector pneoP3300 to get the *CmSlt2* over-expression vector pOVSlt2. The over-expression mutant *OVS-Rac1-1* was generated by introducing pOVSlt2 into Δ*CmRac1-79*, followed by screening for neomycin resistance and qRT-PCR confirmation.

To tag CmSlt2 with the GFP, cDNA of *CmSlt2* without stop codon was amplified by RT-PCR with primer pair 20F/20R (Table S1) and cloned into the *Hin*dIII/*Sma*I sites of pCIT vector. The GFP coding sequence was excised from plasmid pEGFP-1 with *Sma*I/*Bam*HI, and the corresponding fragment was purified. Both the *CmSlt2* and *GFP* coding sequences were cloned into vector pneoP3300 digested with *Xho*I to yield plasmid pSGFP1. Mutants were generated by introducing of pSGFP1 into Δ*CmNox1-6* and the wide-type strain ZS-1, followed by screening for neomycin resistance. GFP signal was observed under a Nikon Eclipse 80i fluorescent microscope (Nikon, Japan).

### DNA extraction and Southern blot analysis

The genomic DNA of the wild-type strain ZS-1 and generated mutants in this study was extracted according to the standard protocols^67^. Southern blot analysis was performed according to Gong *et al.*^16^. Genomic DNA (ZS-1 and CmNox1 mutants) aliquots of 15 μg were digested with *Hin*dIII, separated by electrophoresis on 0.8% agarose gel and transferred onto a Hybond N+ membrane (Amersham Pharmacia Biotech). Interior probe was amplified with the primer pair 30F and 30R (Fig. 2c, Table S1), while the exterior probe was amplified with the primer pair 31F and 31R (Fig. 2c, Table S1). *CmNox2* mutants were also confirmed by Southern blot, with the probe amplified using primer pair 32F and 32R.

### RNA manipulation and qRT-PCR analysis

The total RNA sample of fungal strain was isolated with TriZOL reagent (Invitrogen, USA) according to the manufacturer’s protocols and potential DNA contamination was removed by DNase I treatment (RNase Free) (TaKaRa, Dalian, China). The first-strand cDNA was synthesized with RevertAid™ First Strand cDNA Synthesis Kit (MBI Fermentas, Lithuania) by following the manufacturer’s instructions. In quantitative Real-time PCR, *CmPks1* (primer pair 21F/21R), *CmRac1* (primer pair 22F/22R), *CmNox1* (primer pair 23F/23R), *CmNox2* (primer pair 24F/24R), *CmNoxR* (primer pair 25F/25R), *Cmg1* (primer pair 26F/26R), *CH1* (primer pair 27F/27R) and *CmSlt2* (primer pair 34F/34R) were amplified by using the respective primer pairs (Table S1). As an endogenous control, a 154-bp amplicon of *actin* was amplified with primer pair 28F and 28R (Table S1).

### Yeast two-hybrid system

Yeast two-hybrid analysis was carried out using a GAL4-based yeast two-hybrid system-Matchmaker™ Gold Systems (Clontech, Palo Alto, CA). cDNA of *CmNox1* (1F1/1R1), *CmSlt2* (2F2/2R2), *CmRac1* (3F/3R) and *CmNoxR* (4F/4R), were amplified with the respective primer pairs (Table S1) and inserted into the yeast vector pGADT7. To test the specificity of the interaction, the bait plasmid and the prey plasmids were co-transformed into yeast strain Y2HGold. The transformants were assayed on SD (synthetic dropout)/-Trp-Leu-His-Ade plates and SD/-Trp-Leu-His-Ade plates with X-α-gal for β-galactosidase test.

### Bimolecular fluorescence complementation experiments

For bimolecular fluorescence complementation experiments (BiFC), *CmNox1* and *CmSlt2* were tagged with separated halves of YFP, based on Walter *et al.*^68^, with the following modifications. To fuse YFP-N to *CmSlt2*, *CmSlt2* cDNA without stop codon was amplified with primer pair 7F and 7R and cloned into the *Spe*I/*Sma*I sites of pUC-SPYNE1. To fuse YFP-C to *CmNox1*, *CmNox1* cDNA without stop codon was amplified with primer pair 8F and 8R and cloned into the *Spe*I/*Sma*I sites of pUC-SPYCE1. The two vectors were co-transformed into tobacoo (*Nicotiana benthamiana*). Leaf samples were collected 2 days after agro-infiltration. YFP Fluorescence signal was observed under a Nikon Eclipse 80i fluorescent microscope (Nikon, Japan).

### *In vivo* Co-IP

Agrobacterium strain EHA105 carrying the TrpC-*CmSlt2*-GFP and TrpC-*CmNox1*-Flag expression vectors was coinfiltrated into *C. minitans.* The over-expression mutant was generated by screening for neomycin/hygromycin resistance. Mutant transformed with TrpC-*CmSlt2*-GFP and empty Vector-Flag was generated and used as a control. For *in vivo* Co-IP, 8-day-old mutant total protein was extracted in IP buffer (50 mM Tris pH 7.4, 150 mM NaCl, 1% Triton, 1 mM EDTA, 1 mM MgCl_2_, and protease inhibitor cocktail). A 15 ul aliquot of anti-Flag agarose (Beyotime) was added to the samples, and the mixtures were incubated for 4 h at 4 °C with shaking. The immunoprecipitated proteins were washed three times with IP buffer and eluted with SDS sample buffer. Eluted sample were loaded on the protein gels for immunoblot analysis using anti-GFP antibody or anti-Flag antibody (Sigma).

## Additional Information

**How to cite this article**: Wei, W. *et al.* Nox Complex signal and MAPK cascade pathway are cross-linked and essential for pathogenicity and conidiation of mycoparasite *Coniothyrium minitans. Sci. Rep.* **6**, 24325; doi: 10.1038/srep24325 (2016).

## Supplementary Material

Supplementary Information

## Figures and Tables

**Table 1:** Comparison of hyphal growth rate, conidial production, and parasitic ability among mutants and the wild-type strain ZS-1 of *C. minitans*.

Strains	Growth rate^a^ (mm/d)	Condiation^b^ (x10^7^condia/plate)	Rot index^c^ (%)
ZS-1	2.9 ± 0.1^A^	128 ± 7.2^A^	82 ± 3^A^
Δ*CmNox1-1*	2.8 ± 0.1^A^	0^B^	0^B^
Δ*CmNox1-6*	2.8 ± 0.1^A^	0^B^	0^B^
Δ*CmNox1-107*	2.9 ± 0.1^A^	0^B^	0^B^
*CmNox1-C1*	2.9 ± 0.1^A^	115 ± 4.8^A^	80 ± 2^A^
*CmNox1-C7*	2.8 ± 0.1^A^	119 ± 4.8^A^	81 ± 2^A^
*CmNox1-C8*	2.8 ± 0.1^A^	121 ± 4.8^A^	80 ± 2^A^
Δ*CmNox2-20*	2.9 ± 0.1^A^	120 ± 5.4^A^	81 ± 3^A^
Δ*CmNox2-323*	2.8 ± 0.1^A^	122 ± 5.4^A^	82 ± 3^A^
Δ*CmNox2-347*	2.9 ± 0.1^A^	125 ± 5.4^A^	82 ± 3^A^
Δ*CmRac1-79*	0.5 ± 0.1^B^	0^B^	0^B^
Δ*CmRac1-95*	0.5 ± 0.1^B^	0^B^	0^B^
Δ*CmRac1-102*	0.4 ± 0.1^B^	0^B^	0^B^
*CmRac1-C1*	2.9 ± 0.1^A^	121 ± 9.8^A^	81 ± 4^A^
*CmRac1-C4*	2.9 ± 0.1^A^	119 ± 9.8^A^	80 ± 4^A^
*CmRac1-C7*	2.8 ± 0.1^A^	120 ± 9.8^A^	81 ± 4^A^
*OVS-Rac1-1*	0.6 ± 0.1^B^	0.6 ± 5^A^	40 ± 3^A^
*OVS-Rac1-3*	0.5 ± 0.1^B^	0.6 ± 5^A^	45 ± 3^A^
*OVS-Rac1-4*	0.6 ± 0.1^B^	0.5 ± 5^A^	39 ± 3^A^
Δ*CmNoxR-5*	2.9 ± 0.1^A^	0^B^	0^B^
Δ*CmNoxR-107*	2.8 ± 0.1^A^	0^B^	0^B^
Δ*CmNoxR-160*	2.8 ± 0.1^A^	0^B^	0^B^
Δ*CmBem1-344*	2.9 ± 0.1^A^	124 ± 14.2^A^	84 ± 2^A^
Δ*CmBem1-359*	2.9 ± 0.1^A^	125 ± 14.2^A^	85 ± 2^A^
Δ*CmBem1-370*	2.9 ± 0.1^A^	120 ± 14.2^A^	80 ± 2^A^

^a^Growth rate was detected by measuring the colony diameter of cultures incubatedat 20 °C for 7 days.

^b^Conidia produced by 14-day-old cultures and counted with a haematocytometer.

^c^Rot index of sclerotia was calculated after infected by *C. minitans* for 30 days^14^.

^d^Different letters in the same column indicated statistically significant differences (P = 0.05). Means and standard errors were calculated from three replicates.

## References

[b1] Bolton M., Thomma B. P. H. J. & Nelson B. *Sclerotinia sclerotiorum* (Lib.) de Bary: biology and molecular traits of a cosmopolitan pathogen. Mol Plant Pathol 7, 1–16 (2006). 20507424 10.1111/j.1364-3703.2005.00316.x

[b2] Amselem J. *et al.* Genomic analysis of the necrotrophic fungal pathogens *Sclerotinia sclerotiorum* and *Botrytis cinerea*. PLoS Genet 7, e1002230 (2011). 21876677 10.1371/journal.pgen.1002230 PMC3158057

[b3] Whipps J. M. & Gerlagh M. Biology of *Coniothyrium minitans* and its potential for use in disease biocontrol. Mycol Res 96, 897–907 (1992).

[b4] Li G. *et al.* Biological control of Sclerotinia diseases of rapeseed by aerial applications of the mycoparasite *Coniothyrium minitans*. Eur J Plant Pathol 114, 345–355 (2006).

[b5] Whipps J. M., Sreenivasaprasad S., Muthumeenakshi S., Rogers C. W. & Challen M. P. Use of *Coniothyrium minitans* as a biocontrol agent and some molecular aspects of sclerotial mycoparasitism. Eur J Plant Pathol 121, 323–330 (2008).

[b6] Sandys-Winsch C., Whipps J. M., Gerlagh M. & Kruse M. World distribution of the sclerotial mycoparasite *Coniothyrium minitans*. Mycol Res 97, 1175–1178 (1993).

[b7] Yang L. *et al.* Effects of soil temperature and moisture on survival of *Coniothyrium minitans* conidia in central China. Biol Control 55, 27–33 (2010).

[b8] Paulitz T. C. & Belanger R. R. Biological control in greenhouse systems. Annu Rev of phytopathol 39, 103–33 (2001). 11701861 10.1146/annurev.phyto.39.1.103

[b9] Giczey G., Kernyi Z., Fulop L. & Hornok L. Expression of *cmg1*, an exo-β- glucanase gene from *Coniothyrium minitans*, increase during sclerotial parasitism. Appl Environ Microbiol 67, 865–87 (2001). 11157256 10.1128/AEM.67.2.865-871.2001 PMC92660

[b10] Yang R., Han Y., Li G., Jiang D. & Huang H. Suppression of *Sclerotinia sclerotiorum* by antifungal substances produced by the mycoparasite *Coniothyrium minitans*. Eur J Plant Patho 119, 411–420 (2008).

[b11] Ren L., Li G., Han Y., Jiang D. & Huang H. Degradation of oxalic acid by *Coniothyrium minitans* and its effects on production and activity of beta-1,3-glucanase of this mycoparasite. Biol Control 43, 1–11 (2007).

[b12] Muthumeenakshi S., Sreenivasaprasad S., Rogers C. W., Challen M. P. & Whipps J. M. Analysis of cDNA transcripts from *Coniothyriumminitans* reveals a diverse array of genes involved in key processes during sclerotial mycoparasitism. Fungal Genet Biol 44, 1262–1284 (2008). 17888694 10.1016/j.fgb.2007.07.011

[b13] Zeng F. Y. *et al.* A fungal cell wall integrity-associated MAP kinase cascade in *Coniothyrium minitans* is required for conidiation and mycoparasitism. Fungal Genet Biol 49, 347–357 (2012). 22426009 10.1016/j.fgb.2012.02.008

[b14] Wei W. *et al.* CmPEX6, a gene involved in peroxisome biogenesis, is essential for parasitism and conidiation of sclerotial parasite *Coniothyrium minitans*. Appl Environ Microbiol 79, 3658–3666 (2013). 23563946 10.1128/AEM.00375-13 PMC3675954

[b15] Zeng L. M. *et al.* Degradation of oxalic acid by the mycoparasite *Coniothyrium minitans* plays an important role in interacting with *Sclerotinia sclerotiorum*. Environ Microb 16, 2591–2610 (2014). 10.1111/1462-2920.12409 24467446

[b16] Lou Y. *et al.* CmpacC regulates mycoparasitism, oxalate degradation and antifungal activity in the mycoparasitic fungus *Coniothyrium minitans*. Environ Microb 17(11), 4711–4729 (2015). 10.1111/1462-2920.13018 26278965

[b17] Gong X. Y. *et al.* L-arginine is essential for conidiation in the filamentous fungus *Coniothyrium minitans*. Fungal Genet Biol 44, 1368–1379 (2007). 17897846 10.1016/j.fgb.2007.07.007

[b18] Li B. *et al.* Cyclic GMP as a second messenger in the nitric oxide-mediated conidiation of the mycoparasite *Coniothyrium minitans*. Appl Environ Microbiol 76, 2830–2836 (2010). 20208018 10.1128/AEM.02214-09 PMC2863460

[b19] Qin L. *et al.* Phosphoribosylamidotransferase, the first enzyme for purine *de novo* synthesis, is required for conidiation in the sclerotial mycoparasite *Coniothyrium minitans*. Fungal Genet Biol 48, 956–965 (2011). 21763446 10.1016/j.fgb.2011.06.007

[b20] Scott B. Conservation of fungal and animal nicotinamide adenine dinucleotide phosphate oxidase complexes. Mol Microb 95, 910–913 (2015). 10.1111/mmi.12946 25620385

[b21] Egan M. J., Wang Z. Y., Jones M. A., Smirnoff N. & Talbot N. J. Generation of reactive oxygen species by fungal NADPH oxidases is required for rice blast disease. Proc Natl Acad Sci USA 104, 11772–11777 (2007). 17600089 10.1073/pnas.0700574104 PMC1913907

[b22] Ryder L. S. *et al.* NADPH oxidases regulate septin-mediated cytoskeletal remodeling during plant infection by the rice blast fungus. Proc Natl Acad Sci USA 110, 3179–3184 (2013). 23382235 10.1073/pnas.1217470110 PMC3581893

[b23] Segmuller N. *et al.* NADPH oxidases are involved in differentiation and pathogenicity in *Botrytis cinerea*. Mol Plant Microbe Interact 21, 808–819 (2008). 18624644 10.1094/MPMI-21-6-0808

[b24] Kim H. J., Chen C. B., Kabbage M. & Dickman M. B. Identification and characterization of *Sclerotinia sclerotiorum* NADPH oxidases. Appl Environ Microbiol 77, 7721–7729 (2001). 21890677 10.1128/AEM.05472-11 PMC3209176

[b25] Siegmund U., Marschall R. & Tudzynski P. BcNoxD, a putative ER protein, is a new component of the NADPH oxidase complex in *Botrytis cinerea*. Mol Microbiol 95, 988–1005 (2015). 25402961 10.1111/mmi.12869

[b26] Roca M. G. *et al.* Germling fusion via conidial anastomosis tubes in the grey mould *Botrytis cinerea* requires NADPH oxidase activity. *Fungal* Biol 116, 379–387 (2012). 10.1016/j.funbio.2011.12.007 22385620

[b27] Tanaka A., Christensen M. J., Takemoto D., Park P. & Scott B. Reactive oxygen species play a role in regulating a fungus-perennial ryegrass mutualistic interaction. Plant Cell 18, 1052–1066 (2006). 16517760 10.1105/tpc.105.039263 PMC1425850

[b28] Takemoto D., Tanaka A. & Scott B. A. p67 Phox-like regulator is recruited to control hyphal branching in a fungal-grass mutualistic symbiosis. Plant Cell 18, 2807–2821 (2006). 17041146 10.1105/tpc.106.046169 PMC1626622

[b29] Eaton C. J., Cox M. P. & Scott B. What triggers grass endophytes to switch from mutualism to pathogenism? Plant Sci 180, 190–195 (2011). 21421360 10.1016/j.plantsci.2010.10.002

[b30] Takemoto D. *et al.* Polarity proteins Bem1 and Cdc24 are components of the filamentous fungal NADPH oxidase complex. Proc Natl Acad Sci USA 108, 2861–2866 (2011). 21282602 10.1073/pnas.1017309108 PMC3041104

[b31] Kayano Y., Tanaka A., Akano F. & Scott B. Differential roles of NADPH oxidases and associated regulators in polarized growth, conidiation and hyphal fusion in the symbiotic fungus *Epichloe festucae*. Fungal Genet Biol 56, 87–97 (2013). 23684536 10.1016/j.fgb.2013.05.001

[b32] Lara-Ortiz T., Riveros-Rosas H. & Aguirre J. Reactive oxygen species generated by microbial NADPH oxidase NoxA regulate sexual development in *Aspergillus nidulans*. Mol Microbiol 50, 1241–1255 (2003). 14622412 10.1046/j.1365-2958.2003.03800.x

[b33] Malagnac F., Lalucque H., Lepère G. & Silar P. Two NADPH oxidase isoforms are required for sexual reproduction and ascospore germination in the filamentous fungus *Podospora anserina*. Fungal Genet Biol 41, 982–997 (2004). 15465387 10.1016/j.fgb.2004.07.008

[b34] Cano-Domínguez N., Álvarez-Delfí K., Hansberg W. & Aguirre J. NADPH oxidases NOX-1 and NOX-2 require the regulatory subunit NOR-1 to control cell differentiation and growth in *Neurospora crassa*. Eukaryot Cell 7, 1352–1361 (2008). 18567788 10.1128/EC.00137-08 PMC2519770

[b35] Semighini C. P. & Harris S. D. Regulation of apical dominance in *Aspergillus nidulans* hyphae by reactive oxygen species. Genetics 179, 1919–1932 (2008). 18689883 10.1534/genetics.108.089318 PMC2516069

[b36] Brun S., Malagnac F., Bidard F., Lalucque H. & Silar P. Functions and regulation of the Nox family in the filamentous fungus *Podospora anserina*: a new role in cellulose degradation. Mol. Microbiol 74, 480–496 (2009). 19775249 10.1111/j.1365-2958.2009.06878.x

[b37] Montero-Barrientos M., Hermosa R., Cardoza R. E., Gutierrez S. & Monte E. Functional analysis of the *Trichoderma harzianum* nox1 gene, encoding an NADPH oxidase, relates production of reactive oxygen species to specific biocontrol activity against *Pythium ultimum*. Appl Environ Microbiol 77, 3009–3016 (2011). 21421791 10.1128/AEM.02486-10 PMC3126390

[b38] Xu J. R. MAP kinases in fungal pathogens. Fungal Genet Biol 31, 137–152 (2000). 11273677 10.1006/fgbi.2000.1237

[b39] Zhao X. H., Mehrabi R. & Xu J. R. Mitogen-activated protein kinase pathways and fungal pathogenesis. Eukaryot Cel l6, 1701–1714 (2007). 10.1128/EC.00216-07 PMC2043402 17715363

[b40] Zhao X. H., Kim Y., Park G. & Xu J. R. A mitogen-activated protein kinase cascade regulating infection-related morphogenesis in *Magnaporthe grisea*. Plant Cell 17, 1317–1329 (2005). 15749760 10.1105/tpc.104.029116 PMC1088005

[b41] Rispail N. *et al.* Comparative genomics of MAP kinase and calcium-calcineurin signalling components in plant and human pathogenic fungi. Fungal Genet Biol 46, 287–298 (2009). 19570501 10.1016/j.fgb.2009.01.002

[b42] Wang C. F. *et al.* Functional analysis of the kinome of the wheat scab fungus *Fusarium graminearum*. PLoS Pathog 7, e1002460 (2011). 22216007 10.1371/journal.ppat.1002460 PMC3245316

[b43] Hamel L. P., Nicole M. C., Duplessis S. & Ellis B. E. Mitogen-activated protein kinase signaling in plant-interacting fungi: distinct message from conserved messenger. Plant Cell 24, 1327–1351 (2012). 22517321 10.1105/tpc.112.096156 PMC3398478

[b44] Teichert I. *et al.* PRO40 is a scaffold protein of the cell wall integrity pathway, linking the MAP kinase module to the upstream activator protein kinase C. PLoS Genet 10, e1004582 (2014). 25188365 10.1371/journal.pgen.1004582 PMC4154660

[b45] Turrà D., Segorbe D. & Di Pietro A. Protein kinases in plant-pathogenic fungi: conserved regulators of infection. Annu Rev of Phytopathol 52, 267–288 (2014). 25090477 10.1146/annurev-phyto-102313-050143

[b46] Yun Y. *et al.* The MAPKK FgMkk1 of *Fusarium graminearum* regulates vegetative differentiation, multiple stress response, and virulence via the cell wall integrity and high-osmolarity glycerol signaling pathways. Environ Microbiol 16, 2023–2037 (2014). 24237706 10.1111/1462-2920.12334

[b47] Gu Q., Chen Y., Liu Y., Zhang C. & Ma Z. The transmembrane protein FgSho1 regulates fungal development and pathogenicity via the MAPK module Ste50-Ste11-Ste7 in *Fusarium graminearum*. New Phytologist 206, 315–328 (2015). 25388878 10.1111/nph.13158

[b48] Eaton C. J. *et al.* Disruption of signaling in a fungal-grass symbiosis leads to pathogenesis. Plant Physiol 153, 1780–1794 (2010). 20519633 10.1104/pp.110.158451 PMC2923905

[b49] Chamoun R., Aliferis K. A. & Jabaji S. H. Characterization and transcriptional regulation of *Stachybotrys elegans* mitogen-activated-protein kinase gene smkA following mycoparasitism and starvation conditions. Current Genet 59, 43–54 (2013). 10.1007/s00294-012-0386-2 23271388

[b50] Gruber S. & Zeilinger S. The transcription factor Ste12 mediates the regulatory role of the Tmk1 MAP kinase in mycoparasitism and vegetative hyphal fusion in the filamentous fungus *Trichoderma atroviride*. PLoS One 9, e111636 (2014). 25356841 10.1371/journal.pone.0111636 PMC4214791

[b51] Yang S. L. & Chung K. R. Similar and distinct roles of NADPH oxidase components in the tangerine pathotype of *Alternaria alternate*. Mol Plant Pathol 14, 543–556 (2013). 23527595 10.1111/mpp.12026 PMC6638896

[b52] Medina-Castellanos E., Esquivel-Naranjo E. U., Heil M. & Herrera-Estrella A. Extracellular ATP activates MARK and ROS signaling during injury response in the fungus *Trichoderma atroviride. Front in* Plant Sci 5, 659 (2014). 10.3389/fpls.2014.00659 PMC4240048 25484887

[b53] Jaimes-Arroyo R. *et al.* The SrkA kinase is part of the SakA mitogen-activated protein kinase interactome and regulates stress responses and development in *Aspergillus nidulans*. Eukaryot Cel l 14, 495–510 (2015). 10.1128/EC.00277-14 PMC4421008 25820520

[b54] Lambou K. *et al.* The crucial role of the Pls1 tetraspanin during ascospore germination in *Podospora anserine* provides an example of the convergent evolution of morphogenetic processes in fungal plant pathogens and saprobes. Eukaryot Cell 7, 1809–1818 (2008). 18757568 10.1128/EC.00149-08 PMC2568061

[b55] Dirschnabel D. E. *et al.* New insights into the roles of NADPH oxidases in sexual development and ascospore germination in *Sordaria macrospora*. Genetics 196, 729–744 (2014). 24407906 10.1534/genetics.113.159368 PMC3948803

[b56] Schurmann J., Buttermann D. & Herrmann A. Molecular characterization of the NADPH oxidase complex in the ergot fungus *Claviceps purpurea*: CpNox2 and CpPls1 are important for a balanced host-pathogen interaction. Mol Plant Microbe Interact 26, 1151–1164 (2013). 23777432 10.1094/MPMI-03-13-0064-R

[b57] Mahlert M., Levelek L., Hlubek A., Sandrock B. & Bolker M. Rac1 and Cdc42 regulate hyphal growth and cytokinesis in the dimorphic fungus *Ustilago maydis*. Mol Microbiol 59, 567–578 (2006). 16390450 10.1111/j.1365-2958.2005.04952.x

[b58] Chen J. *et al.* Rac1 is required for pathogenicity and Chm1-dependent conidiogenesis in rice fungal pathogen *Magnaporthe grisea*. PLoS Pathog 4, e1000202 (2008). 19008945 10.1371/journal.ppat.1000202 PMC2575402

[b59] Rolke Y. & Tudzynski P. The small GTPase Rac and the p21- activated kinase Cla4 in *Claviceps purpurea*: interaction and impact on polarity, development and pathogenicity. Mol Microbiol 68, 405–423 (2008). 18284596 10.1111/j.1365-2958.2008.06159.x

[b60] Li H. Y. *et al.* The small GTPase RacA mediates intracellular reactive oxygen species production, polarized growth, and virulence in the human fungal pathogen *Aspergillus fumigatus*. Eukaryot Cell 10, 174–186 (2011). 21183690 10.1128/EC.00288-10 PMC3067399

[b61] Zhang C. *et al.* Functional characterization of Rho family small GTPases in *Fusarium graminearum*. Fungal Genet Biol 61, 90–99 (2013). 24055721 10.1016/j.fgb.2013.09.001

[b62] Hope H., Schmauch C., Arkowitz R. A. & Bassilana M. The *Candida albicans* ELMO homologue functions together with Rac1 and Dck1, upstream of the MAP Kinase Cek1, in invasive filamentous growth. Mol Microbiol 76, 1572–1590 (2010). 20444104 10.1111/j.1365-2958.2010.07186.x

[b63] Lalucque H., Malagnac F., Brun S., Kicka S. & Silar P. A Non-mendelian MAPK-generated hereditary unit controlled by a second MAPK pathway in *Podospora anserine*. Genetics 191, 419–433 (2012). 22426880 10.1534/genetics.112.139469 PMC3374308

[b64] Bidard F., Coppin E. & Silar P. The transcriptional response to the inactivation of the PaMpk1 and PaMpk2 MAP kinase pathways in *Podospora anserine*. Fungal Genet Biol 49, 643–652 (2012). 22721649 10.1016/j.fgb.2012.06.002

[b65] Cheng J. S. *et al.* Production, survival and efficacy of *Coniothyrium minitans* conidia produced in shaken liquid culture. FEMS Microbiol Lett 227, 127–131 (2003). 14568158 10.1016/S0378-1097(03)00666-9

[b66] Xie J. *et al.* Characterization of debilitation-associated mycovirus infecting the plant-pathogenic fungus *Sclerotinia sclerotiorum*. J Gen Virol 87: 241–249 (2006). 16361437 10.1099/vir.0.81522-0

[b67] Sambrook J. & Russell D. W. Molecular cloning: A laboratory manual. Cold Spring Harbor Laboratory Press, Cold Spring Harbor, NY. (2001).

[b68] Walter M. *et al.* Visualization of protein interactions in living plant cells using bimolecular fluorescence complementation. Plant J 40, 428–438 (2004). 15469500 10.1111/j.1365-313X.2004.02219.x

