# Supplementary material for: Nox Complex signal and MAPK cascade pathway are cross-linked and essential for pathogenicity and conidiation of mycoparasite Coniothyrium minitans
Source: Sci Rep. 2016 Apr 12;6:24325. doi: 10.1038/srep24325 (PMC4828707; doi:10.1038/srep24325)

**Title: Nox Complex signal and MAPK cascade pathway are cross-linked and essential for pathogenicity and conidiation of mycoparasite *Coniothyrium minitans***

Wei Wei^1, 2^﹡, Wenjun Zhu^1, 2^, Jiasen Cheng^2^, Jiatao Xie^2^, Daohong Jiang^1, 2^, Guoqing Li^1, 2^, Weidong Chen^3^, Yanping Fu^2^

^1^ State Key Laboratory of Agricultural Microbiology, Huazhong Agricultural University, Wuhan 430070, Hubei Province, P R China.

^2^ The Provincial Key Lab of Plant Pathology of Hubei Province, College of Plant Science and Technology, Huazhong Agricultural University, Wuhan, 430070, Hubei Province, P R China.

^3^ United States Department of Agriculture, Agricultural Research Service, Washington State University, Pullman, WA, USA.

﹡Present address: Institute for Interdisciplinary Research, Jianghan University, Wuhan 430056, Hubei Province, P R China.

Dr. Yanping Fu, Professor

Plant Pathology

College of Plant Science and Technology

Huazhong Agricultural University

Wuhan, 430070, Hubei Province

P R China

Tel: 86-27-87280487; Fax: 86-27-87397735

E-mail: yanpingfu@mail.hzau.edu.cn

Main text (excluding the references, table footnotes, and figure legends): 4683 words; Figures, 7; Tables, 1

**Supplementary Table S1.** Primer used for vector construction and PCR.

| Primers used for Yeast two-hybrid |
| --- |
| 1F1: 5’ GCCCGGGTATGGCGGGCGGCGGATCAGTATG 3’  1R1: 5’ CCCGGGCTAGAAATGCTCCTTCCAAAAC 3’  2F1: 5’ CGGAATTC ATGGACAAAACTGGCTACGCAG 3’  2R1: 5’ CGGGATCC CTAGAAGTTCTCCTTGCCCCACAC 3’  2F2: 5’ CGGAATTCATGTCTGACCTCGCCGGCCGC 3’  2F2: 5’ CGGGATCCCCGCATTCGAGCGTCCAGTCCCTGC 3’  3F: 5’ CCCATATGATGGCTGCACCCACCACCCAG 3’  3R: 5’ GCGTCGACCTTGCTCTTCTTGACCTTGCCG 3’  4F: 5’CCCATATGATGTCTCTGAAGCAGGAAATCG 3’  4R: 5’CGGAATTCCTAGTATTGGGAGACCGAGAAGG 3’ |
| Primers used for GFP-Slt2 fusion and BiFC |
| 5F: 5’ AAGCTTATGTCTGACCTCGCCGGCCGC 3’  5R: 5’ CCCGGGCCGCATTCGAGCGTCCAGTCCCTGC 3’  6F: 5’ CCCGGGATGGTGAGCAAGGGCGAGGAGC 3’  6R: 5’ GGATCCCTTGTACAGCTCGTCCATGCCG 3’  7F: 5’ ACTAGTATGTCTGACCTCGCCGGCCGC 3’  7R: 5’ GGTACCTTTGTCGTCGTCGTCTTTGTAGTCC 3’  8F: 5’ GGTACCATGGCGGGCGGCGGATCAGTATG 3’  8R: 5’ CCCGGGGAAATGCTCCTTCCAAAACTTG 3’ |
| Primers used for replacement vectors construction |
| 9F: 5’ GAGCTCTTCTTCGTCGTTGTCTTGGT 3’  9R: 5’ GCTCTAGACTGGTGGAAGCGGAATACA 3’  10F: 5’ GCTCTAGATGCCGAGCAGAAATGGGATA 3’  10R: 5’ CTCGAGCCTCCGCTCCCTCCTACATC 3’  11F: 5’ CGAAGCTTATGCTGCTTGTCCGCACTAC 3’  11R: 5’ CGGTCGACCCACCGCATCAGAACCCATC 3’  12F: 5’ GCTCTAGAGGGTGCTGAATGACCTTGGA 3’  12R: 5’ GGGGTACCTTGCCCACTTTCAGCAGGAT 3’  13F: 5’ GCTCTAGAGGAAGTCGCAACGGCTACGG 3’  13R: 5’ GGGGTACCGTGGTGAGTGCGTGGCGTAT 3’  14F: 5’ CGAAGCTTGAGCCGCATCACCGTAGTCAC 3’  14R: 5’ CGGTCGACGCCAACGGGAATGGAGAAGA 3’  15F: 5’ GCGTCGACGACAATCCTGCGAACAATCC  15R: 5’ GCTCTAGATATGGCGAGGCGGAAGAAGC  16F: 5’ GCTCTAGACCGTTCTCAGCCCTCGTCCAC  16R: 5’ CGAGCTCCTTGTAACTGTCCGTTCCTG  17F: 5’ GAGCTCCTTTCCGCCTTCCATTGTCTG 3’  17R: 5’ TCTAGACGGTCGCTCTGCCTTGAAAT 3’  18F: 5’ TCTAGACCAGGACCAACGAGCATC 3’  18R: 5’ CTCGAGGTCGTGAGTGCCGAAGAACC 3’ |
| Primers used for complementary and over-expression vectors construction |
| 19F: 5’ GGGGTACCGCTCTCCGTGGTTCCTCCGCATCTC 3’  19R: 5’ GGGGTACCCCGCTTCCACCAGCCACCAGCCGAC 3’  20F: 5’ AAGCTTATGTCTGACCTCGCCGGCCGC 3’  20R: 5’ CCCGGGCCGCATTCGAGCGTCCAGTCCCTGC 3’ |
| Primers used for qRT-PCR |
| 21F: 5’CGCTGAGGTGAACAAGAGGC 3’  21R: 5’ GGACCACTTCTCGGGACACTT 3’  22F:5’CGGACAGGAAGATTACGACC 3’  22R: 5’CTTGTTGCCGCCGAGGATAA 3’  23F: 5’ CAGGAGGTGATGGTGAAGGG 3’  23R: 5’TCGTCAAGGTTGTCCGCCAC 3’  24F:5’ CAGATGCTGAGCCGAAAGGA3’  24R: 5’CGTCCGTATGGTTGGTGATT 3’  25F:5’ CAGAGCAGAGCACGACAGCA 3’  25R:5’ GAACAGATTGAGCACGGGACA 3’  26F: 5’GTTGCGGTGAGGGATGTGAC 3’  26R: 5’ CGTTGCCACCCTCAATGTAA 3’  27F: 5’ GGTATGTGGTGGGAAAGCAGC 3’  27R: 5’ TGGCAGACGAGGTCGCAAGAT 3’  28F: 5’ GTCCGTGACATCAAGGAGAAGC 3’  28R: 5’ TTGCCAATGGTGATGACCTGAC 3’  29F: 5’ ACCGTGAGAAGATGACCC 3’  29R: 5’ AAGGACAGAAGGCTGGAAG 3’ |
| Primers used for Southern bolt analysis |
| 30F: 5’ GCTTAGACTGCGACATACCCG 3’  30R: 5’ TCGTCAAGGTTGTCCGCCAC 3’  31F: 5’ GGTATTGCCATGAGCCATAACG 3’  31R: 5’TGTAGTCTTTGCCAGTACCACACTC 3’  32F: 5’ GGGTGCTGAATGACCTTGGA 3’  32R: 5’TTGCCCACTTTCAGCAGGAT 3’ |

**Supplementary Figure S1．Phylogenetic relationships of CmNox1 and CmNox2.** The amino acid sequences of Nox1/Nox2 from *Coniothyrium minitans*, *Alternaria alternate*, *Curvularia lunata*, *Pyrenophora tritici-repentis*, *Neofusicoccum parvum*, *Coniosporium apollinis*, *Sclerotinia borealis*, *Sclerotinia sclerotiorum*, F*usarium oxysporum*, *Ustilaginoidea virens*, *Verticillium dahliae*, and *Colletotrichum gloeosporioides*, were analyzed by MEGA version 4.


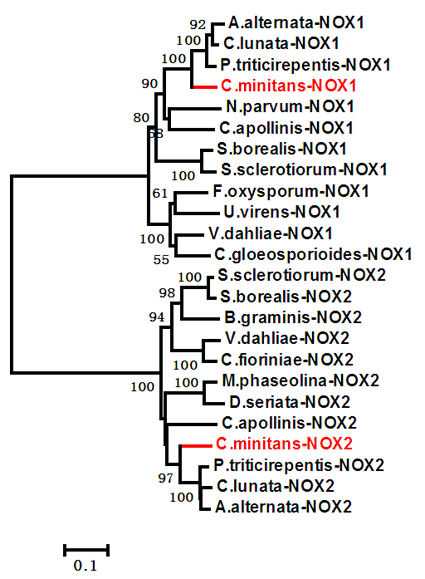


**Supplementary Figure S2. Construction of replacement vector and confirmation of *CmNox1* mutants.** (a) Strategic map of the *CmNox1* replacement construct pNox1-3300 and pNox2-3300. The *hph* gene cassette was cloned into the corresponding sites of vector pNox1-3300 or pNox2-3300 to replace the 1200-bp of the *CmNox1* ORF or 887-bp of the *CmNox2* ORF. (b) Total RNA samples extracted from mycelia of ZS-1, △CmNox1-1, △CmNox1-6, CmNox1-C3 and CmNox1-C8 were subjected to RT-PCR using *CmNox1* gene-specific primers 2F and 2R (Table 2). The RT-PCR product is a 300-bp fragment in ZS-1, CmNox1-C3 and CmNox1-C8 as predicted, but is missing in the deletion mutant△CmNox1-1 and △CmNox1-6. (c) Southern blot analysis of mutants. Genomic DNA (15 μg per lane) of ZS-1, △CmNox1-1, △CmNox1-6 or CmNox1-C8 was digested with *Hin*dIII. The same filter was hybridized with a probe corresponding to the hygromycin resistance cassette (hph) (P1) and then with a probe derived from the replaced fragment of *CmNox1* (P2).


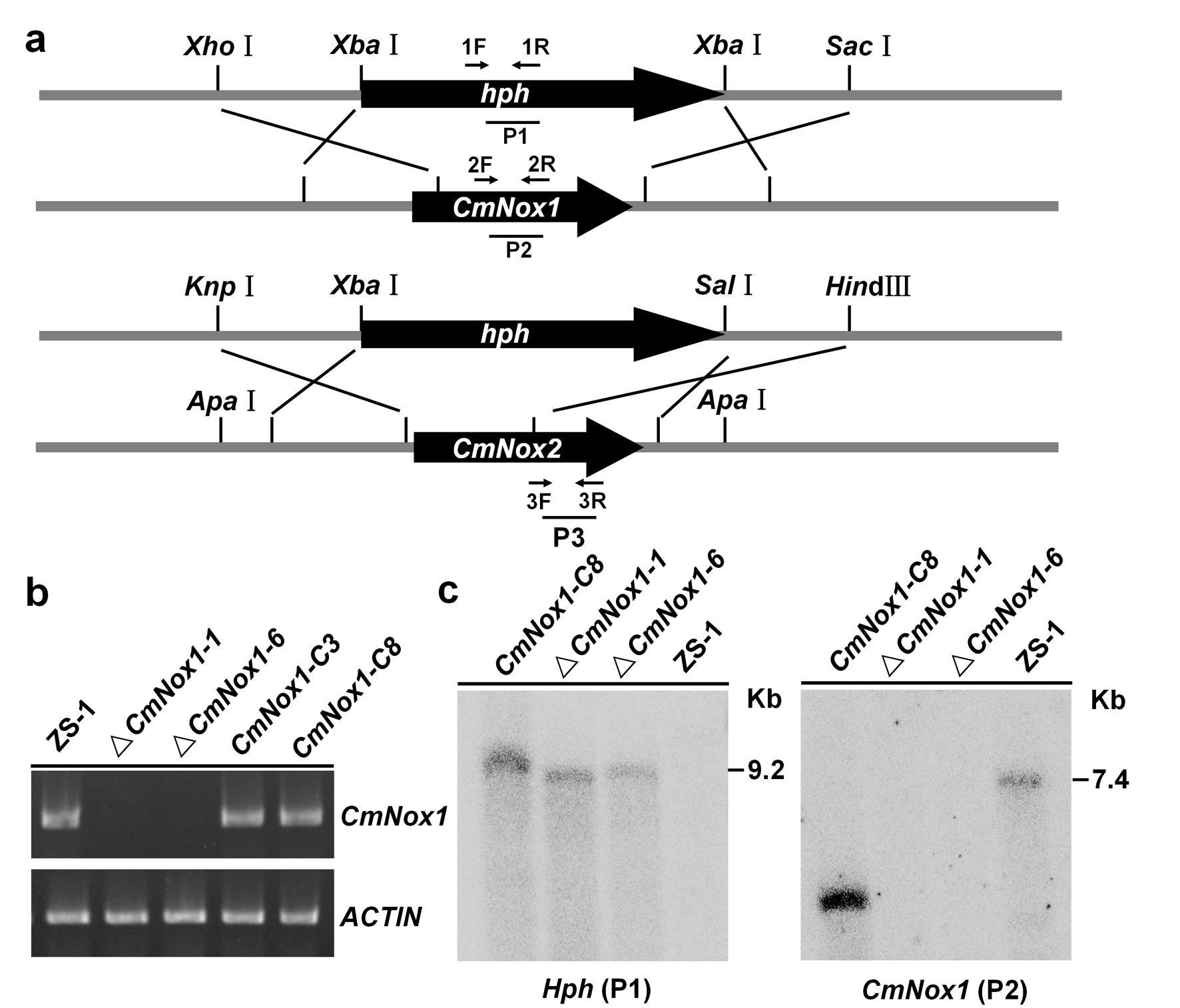

Supplement: Supplementary Information [file srep24325-s1.doc]
